# Nurses’ perception on mental health interventions in Primary Health Care

**DOI:** 10.1590/0034-7167-2024-0260

**Published:** 2025-09-08

**Authors:** Rafaela Sales Medeiros, Divane de Vargas, Jaqueline Lemos de Oliveira

**Affiliations:** I Universidade de São Paulo. São Paulo, São Paulo, Brazil

**Keywords:** Primary Health Care, Nursing, Mental health, Psychiatry, Psychosocial Intervention, Atención Primaria de Salud, Enfermería, Salud Mental, Psiquiatría, Intervención Psicosocial

## Abstract

**Objectives:**

to verify the perception of Primary Health Care nurses regarding mental health interventions used in their daily work.

**Methods:**

quantitative, descriptive-exploratory study, developed in 66 Primary Health Care Units. The sample consisted of 239 nurses. For the descriptive analysis, according to the interventions recommended in the national guidelines and systematic reviews, the practices were classified into three components: universal practice (I), specifically in mental health (II) and educational and universal practices (III).

**Results:**

regarding component I, most participants considered all items as being within their attribution and possible to perform, only for the item Integrative and Complementary Practices, most participants did not consider themselves qualified. Among the specific mental health activities, the items therapeutic interpersonal relationships, advanced practices in rehabilitation, therapeutic groups and application of scales were those for which nurses considered themselves least qualified to perform. Regarding component III, continuing education, this was the item for which only 40% of nurses considered themselves qualified to perform.

**Conclusions:**

it was found that nurses recognize mental health practices in their daily service, as well as perform them and understand them as being within the scope of their actions; however, there is still the perception that they do not feel qualified to perform such practices.

## RESUMEN

**Objetivos:**

evaluar la percepción de los enfermeros de la Atención Primaria de Salud sobre las intervenciones en salud mental utilizadas en su trabajo diario.

**Métodos:**

estudio cuantitativo, descriptivo-exploratorio desarrollado en 66 Unidades Básicas de Salud. La muestra incluyó 239 enfermeros. Para el análisis descriptivo, de acuerdo con las intervenciones recomendadas en las guías nacionales y revisiones sistemáticas, las prácticas se clasificaron en tres componentes: práctica universal (I), práctica específica en salud mental (II) y prácticas educativas y universales (III).

**Resultados:**

respecto al componente I, la mayoría de los participantes consideraron todos los ítems como de su competencia y posibles de realizar, sólo para el ítem Prácticas Integrativas y Complementarias, la mayoría de los participantes no se consideraron capaces. Entre las actividades específicas de salud mental, los para los cuales los enfermeros se consideraron menos capacitados para realizar los siguientes ítems; relaciones interpersonales terapéuticas, prácticas avanzadas de rehabilitación, grupos terapéuticos y aplicación de escalas. Respecto al componente III, la formación continua fue el ítem para el cual sólo el 40% de los enfermeros se consideraron capacitados para llevarlo a cabo.

**Conclusiones:**

los enfermeros reconocen las prácticas de salud mental en la atención diaria del servicio, las realizan y las entienden como dentro del alcance de su actuación. Sin embargo, aún existe la percepción de que no se sienten capacitados para realizar dichas prácticas.

## INTRODUCTION

Data from the World Health Organization (WHO) estimate that approximately 720 million people have mental health-related needs, such as mental, neurological, and alcohol and drug use disorders, with these conditions being responsible for disabilities and a large burden of disease worldwide^(1)^. In Brazil in 2019, mental disorders (MD) caused 22% of disability-adjusted life years (DALYs) and 38% of all years lived with disability (YLDs), with the most common disorders responsible for these losses being depressive disorders, anxiety disorders, bipolar disorder, and schizophrenia^(2)^. In addition, the country ranks fourth among Latin American countries with the highest annual increase in suicides^(3)^.

According to the WHO, 20% of Primary Health Care (PHC) visits are related to MDs, which reinforces the importance of these settings and the professionals who work there for the mental health care of the population^(4)^. Contrary to epidemiological indicators, there is evidence of a shortage of specialized professionals to care for mental health in Brazil and around the world, which, combined with the scarcity of actions and lack of training of professionals, including nurses, devalues the service to address this scenario^(5, 6, 7)^.

Among the reasons indicated in the literature for the difficulties of PHC nurses in engaging with mental health demands in these scenarios are: the absence of strategies and instruments for care in the area, the lack of training and insecurities in dealing with specific situations in daily practice^(5, 6, 7, 8)^.

These data result in limited action by nurses in providing care and identifying symptoms of mental illness, which directly interferes with care practice, which ultimately directs care to other professionals on the team or to specialized services, without the use of mental health interventions that are specific to nursing professionals and are already recommended in national guidelines and professional regulations^(5, 6, 7, 8)^.

## OBJECTIVES

To verify the perception of PHC nurses regarding mental health interventions used in their daily work.

## METHODS

### Ethical aspects

This study was approved by the Ethics and Research Committee of the host institution. The signature for the Informed Consent Form was obtained from all subjects.

### Study design, period and setting

Descriptive exploratory study with a cross-sectional design guided by the STROBE tool. Carried out between March and August 2023 in traditional Primary Health Care Units (PHCU) (without Family Health Strategy) in the city of São Paulo^(9)^.

### Population and sample

In order to recruit a representative sample of nurses and minimize selection bias, stratified sampling in homogeneous subgroups was used. The sample calculation considered a margin of error of 5%; refusals and/or losses of approximately 10%; confounding control factors of 20% and a confidence level of 95%, which resulted in a minimum sample of 199 nurses; the final sample consisted of 239 nurses. The inclusion criteria were to have been a nurse in the unit for a minimum period of 6 months (justified as a period of adaptation and integration of the professional to the team and to the experiences related to the health-disease-illness process of the users) and to have already treated people with needs related to mental health. The exclusion criterion was to have any chronic health condition that would prevent participation in the research.

### Data Collection

For data collection, an instrument created by the authors was used, with multiple-choice questions with simple and objective answers, to minimize response bias, distributed in three categories: I - identification and sociodemographic data (15 questions); II - profile of academic and care training (14 questions). In this item, the frequency of care for people with complaints related to persistent disorders, such as depression, anxiety and schizophrenia, as well as common mental disorders, characterized by non-psychotic somatic symptoms that generate functional incapacity, such as sadness, fatigue, discouragement, changes in sleep and appetite, pain, dizziness or even gastric and intestinal disorders^(10)^ was questioned; III - Questionnaire regarding the nurse’s performance (20 questions). This last category includes interventions selected from Primary Care Notebook 34, prepared by the Health Care Secretariat, in partnership with the Ministry of Health, which shares specific knowledge and presents tools and strategies for therapeutic interventions for mental health care^(11)^; COFEN resolution 599/2018, which provides for general and specific mental health actions that are the responsibility of nurses^(12)^; and a scoping review that describes nursing interventions in mental health in PHC^(6)^, highlighting the importance of appropriating these tools for care.

The categories were classified into three components developed based on the primary care guidelines for teamwork^(9)^, namely: Component I - Universal practice interventions, covering interventions that interface with other lines of PHC units; Component II - Specific practice interventions for mental health and psychiatry; and Component III - Interventions based on educational and managerial activities, related to preparing the nursing team to act in mental health situations and recording. These categories supported the interpretation of the data.

In the city of São Paulo, during the collection period, there were 392 registered PHCU, distributed across six Regional Health Coordination Offices (RHCO). To meet the regional sample requirements, units were selected according to the availability of both managers and data collection staff, including 17 units in the Southeast RHCO; 2 units in the Center RHCO; 16 units in the East RHCO; 12 units in the North RHCO; 8 units in the West RHCO and 11 units in the South RHCO, totaling 66 PHCU. Interviews were scheduled in advance and data collection took place in person at the participants’ workplaces. Data were stored and managed using forms from the REDCap electronic data capture tool. The average length of interviews was 25 minutes.

### Data analysis

For data analysis, R Development Core Team, version 4.1.0, was used, where frequency distribution tables were systematized to present the findings, and Simples and descriptive statistical analysis were applied.

## RESULTS

### Sociodemographic and professional characterization of the sample

The sample consisted of 239 nurses, mostly female (88.2% n=211); with a mean age of 40 years (SD 11.2); married (55.6% n=133); with 1 to 4 years of experience in the unit (45.4% n=108) and full-time work (75.3% n=180). The mean time since graduation was 12 years (SD = 5.25) and the majority were from private institutions (82.4% n=197). Regarding educational level, 95.2% (n=210) had completed a specialization course, and the most frequent specialty was family health strategy (39.4% n=86), followed by public health (38.9% n=85). Regarding preparation to work in mental health and psychiatry, the majority of interviewees (91.6% n=164) reported only preparation in undergraduate studies, offered through lectures.

### Frequency of mental health care

**Figure 1:**
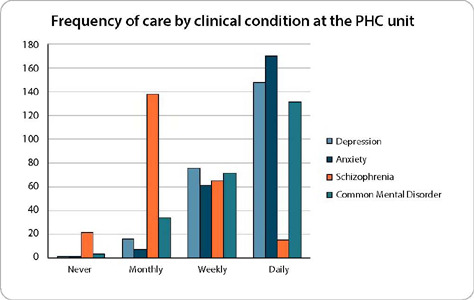
Frequency of care by clinical condition at the PHC unit

When asked about the frequency of mental health care, anxiety (71%) and depression (61.5%) were the most reported by nurses, followed by common mental disorders treated daily by 54.8% of nurses (Figure 1).

### Component I - Universal Practice Interventions

Among the universal practices, those aimed at all users seeking health care in PHC, including people with mental health-related needs, the nurses’ responses related to the recognition of attribution, the possibility of implementation, and the perception of qualification are shown in Table 1.

**Table 1:** Distribution of participants’ responses to component I of universal care practices regarding recognition as a practice under their responsibility, possibility of implementation, and perception of their own qualification for use in daily practice in the Primary Health Care setting, São Paulo, São Paulo, Brazil, 2023 (N=239)

Intervention	Consider it your responsibility	n(%)	Possible to perform in the PHC setting	n(%)	Consider yourself qualified	n(%)
Counselling	Yes	230(96.6)	Yes	224(93.7)	Totally	134(56)
	No	6(2.5)	No	9(3.7)	Partially	93(38.9)
	I’m not sure	2(0.8)	I’m not sure	6(2.5)	Do not consider	12(5.0)
Screening	Yes	209(95.0)	Yes	219(99.5)	Totally	189(85.9)
	No	10(4.5)	No	1(0.4)	Partially	30(13.6)
	I’m not sure	1(0.4)	I’m not sure	19(7.9)	Do not consider	1(0.4)
Nursing Consultation	Yes	223(93.3)	Yes	217(90.8)	Totally	153(64)
	No	9(3.7)	No	13(5.4)	Partially	72(30.1)
	I’m not sure	7(2.9)	I’m not sure	9(3.7)	Do not consider	14(5.8)
Prenatal care	Yes	228(95.4)	Yes	224(93.7)	Totally	150(62.7)
	No	7(2.9)	No	6(2.5)	Partially	83(34.7)
	I’m not sure	4(1.6)	I’m not sure	9(3.7)	Do not consider	6(2.5)
Home care	Yes	211(88.2)	Yes	206(86.1)	Totally	131(54.8)
	No	17(7.1)	No	18(7.5)	Partially	91(38.0)
	I’m not sure	11(4.6)	I’m not sure	15(6.2)	Do not consider	17(7.1)
Integrative and complementary practices	Yes	181(75.7)	Yes	205(85.7)	Totally	56(23.4)
	No	45(18.8)	No	25(10.4)	Partially	54(22.5)
	I’m not sure	13(5.4)	I’m not sure	9(3.7)	Do not consider	129(53.9)
Case management	Yes	216(90.3)	Yes	223(93.3)	Totally	142(59.4)
	No	16(6.6)	No	11(4.6)	Partially	82(34.3)
	I’m not sure	7(2.9)	I’m not sure	5(2.0)	Do not consider	15(6.2)

### Component II - Specific mental health and psychiatric practice interventions

Among the specific mental health practices related to the care of people with mental health-related needs, the nurses’ responses regarding the recognition of their role, the possibility of implementation and the perception of their qualification are shown in Table 2.

**Table 2:** Distribution of participants’ responses to component II of specific Mental Health and Psychiatry practice regarding the recognition as a practice under their responsibility, with the possibility of implementation and perception of their qualification for use in daily practice in the Primary Health Care setting, São Paulo, São Paulo, Brazil, 2023 (N=239)

Intervention	Consider it your responsibility	n(%)	Possible to perform in the PHC setting	n(%)	Consider yourself qualified	n(%)
Counseling	Yes	212(88.7)	Yes	222(88.7)	Totally	121(50.6)
	No	21(8.7)	No	9(3.7)	Partially	104(43.5)
	I’m not sure	6(2.5)	I’m not sure	6(2.5)	Do not consider	14(5.8)
Therapeutic Interpersonal Relationship	Yes	191(79.9)	Yes	182(76.4)	Totally	80(33.4)
	No	28(11.7)	No	39(16.3)	Partially	111(46.4)
	I’m not sure	20(8.37)	I’m not sure	17(7.14)	Do not consider	48(20.0)
Social support	Yes	227(94.9)	Yes	219(91.6)	Totally	143(60.0)
	No	11(4.6)	No	10(4.1)	Partially	83(34.8)
	I’m not sure	1(0.4)	I’m not sure	10(4.1)	Do not consider	12(3.3)
Family support	Yes	212(88.7)	Yes	211(88.2)	Totally	137(57.3)
	No	15(6.8)	No	20(8.3)	Partially	85(35.5)
	I’m not sure	12(5.0)	I’m not sure	8(3.3)	Do not consider	17(7.1)
Advanced Practices in Rehabilitatio	Yes	163(68.2)	Yes	172(71.9)	Totally	66(27.6)
	No	61(25.5)	No	49(20.5)	Partially	87(36.4)
	I’m not sure	15(6.2)	I’m not sure	18(7.5)	Do not consider	86(35.9)
User safety related to the use of psychotropic drugs	Yes	187(78.2)	Yes	209(87.4)	Totally	128(53.5)
	No	39(16.3)	No	19(7.9)	Partially	79(33.0)
	I’m not sure	13(5.4)	I’m not sure	11(4.6)	Do not consider	32(13.3)
Referrals and matrix support	Yes	219(91.6)	Yes	232(97.0)	Totally	154(64.7)
	No	12(5.0)	No	4(1.6)	Partially	76(31.9)
	I’m not sure	8(3.3)	I’m not sure	3(1.2)	Do not consider	8(3.3)
Singular Therapeutic Project	Yes	217(91.1)	Yes	212(97.0)	Totally	137(57.3)
	No	13(5.4)	No	17(7.1)	Partially	80(33.4)
	I’m not sure	8(3.3)	I’m not sure	10(4.1)	Do not consider	22(9.2)
Therapeutic groups	Yes	178(74.4)	Yes	201(84.1)	Totally	66(27.6)
	No	44(18.4)	No	23(9.6)	Partially	118(49.3)
	I’m not sure	17(7.1)	I’m not sure	15(6.2)	Do not consider	55(23.0)
Scales’ application	Yes	169(70.7)	Yes	181(75.7)	Totally	80(33.4)
	No	45(18.8)	No	35(14.6)	Partially	69(28.8)
	I’m not sure	25(10.4)	I’m not sure	23(9.6)	Do not consider	90(37.6)

### Component III - Interventions based on educational and managerial activities

Among the educational and managerial activities, the nurses’ responses related to the recognition of attribution, the possibility of realization and the perception of training are evidenced in Table 3.

**Table 3:** Distribution of participants’ responses to component III - Interventions based on educational and managerial activities regarding recognition as a practice under their responsibility, possibility of implementation and perception of their own qualification for use in daily practice in the Primary Health Care setting, São Paulo, São Paulo, Brazil, 2023 (N=239)

Intervention	Consider it your responsibility	n(%)	Possible to perform in the PHC setting	n(%)	Consider yourself qualified	n(%)
Health Education	Yes	211(88.2)	Yes	222(92.8)	Totally	125(52.3)
	No	21(8.7)	No	12(5.0)	Partially	89(37.2)
	I’m not sure	7(2.9)	I’m not sure	5(2.0)	Do not consider	25(10.4)
Continuing Education	Yes	208(87.0)	Yes	207(86.6)	Totally	96 (40.1)
	No	23(9.6)	No	24(10.0)	Partially	103(43.1)
	I’m not sure	8(3.3)	I’m not sure	8(3.3)	Do not consider	40(16.7)
Record	Yes	233(97.4)	Yes	235(98.3)	Totally	199(83.2)
	No	5(2.0)	No	3(1.2)	Partially	34(14.2)
	I’m not sure	1(0.4)	I’m not sure	1(0.4)	Do not consider	6(2.5)

## DISCUSSION

The results of the study suggest that nurses recognize mental health interventions and identify them as activities under their responsibility, serving, in their practice, people with mental health-related needs and implementing the interventions described; feasibility is also reported in the implementation as possible in PHC. This result reflects the role of nurses as relevant in mental health care, as they are the frontline professionals whose work goes beyond physical care, integrating biopsychosocial, economic and environmental spheres, in order to ensure effective and comprehensive care to the individual^(7)^. These are essential aspects, considering PHC, which is based on the care model centered on the person, the family, the establishment of bonds, comprehensiveness and longitudinality, which surpasses the biomedical model^(13, 14, 15)^.

Regarding component I, consisting of universal activities, most participants considered all items as being within their responsibility and possible to perform in the PHC setting; however, regarding the item Integrative and Complementary Practices (ICPS), most did not consider themselves qualified to perform such intervention. The Federal Nursing Council (Cofen), through Resolution 739/24, regulated the role of nursing in ICPS. In this sense, properly trained professionals have autonomy to act with such interventions^(16)^. Since this is a recent resolution, it is understood that it is necessary for services to encourage nursing professionals to become trained and use these interventions as care strategies in PHC, since they stimulate the comprehensive and multidisciplinary perspective of care, by enabling greater patient adherence to treatments^(17)^. However, it is worth noting that the availability of both human and material resources are items that limit the expansion of access to this care, in view of the results of a previous study^(18)^. In this sense, these are factors that should be considered in the implementation of policies that encourage such care.

It is noteworthy that reception was the item that most professionals identified as being within their attribution. A review study indicates that this intervention is the main technology of care and is within the list of skills of nursing professionals^(6)^. It is understood that it is a relational intervention, essential for creating bonds, which promotes comprehensive and targeted care^(15)^. Considering mental health care, this action facilitates the identification of signs, symptoms and possible triggers of the disorder and, consequently, the direction of the appropriate flow of care^(19)^. Reception is described in the guidelines and in the functions of nurses in national guidelines^(9, 12)^, and is therefore an important tool for mental health care in PHC^(20)^.

According to PHC guidelines, reception and triage are important factors in the process of holding the team accountable for the user. In this sense, the use of qualified listening and the search for comprehensive care ensure resolute and responsible care. The developments observed during nursing consultations that favor user care are enhanced with individualized care, by promoting actions coordinated with the multidisciplinary team for prevention, risk signaling and health promotion^(8, 21, 22)^. Authors point to training as a tool for quality care, as well as the need for clear protocols to guide practice^(23, 24, 25)^.

The nurse’s work process is marked by the accumulation of care tasks (care for users, families and the community in individual and collective contexts) with administrative tasks (people management and administrative processes). This cluster of tasks is marked by the conflict between providing direct care and carrying out administrative processes, and the demands related to fulfilling administrative activities are not equivalent to those related to the quality of health care for users and families^(23)^. If, on the one hand, this accumulation of tasks can harm user care, on the other hand, it is understood that, as an important professional in multidisciplinary work in PHC, this articulation is fundamental in the transition of care to specialized services, since, in the context of case management, it articulates the managerial and care dimensions to identify health issues, promote assertive actions and guide care^(13, 14, 26, 27, 28)^.

Regarding the activities of component II, most participants considered all of them to be within their remit and possible to perform in PHC. However, the activities of Therapeutic Interpersonal Relationships (TIR), the implementation of therapeutic groups and the application of scales were reported by more than half of the nurses with little or no training for the exercise. It is worth noting that the TIR is based on the concepts of Hildegard Peplau’s Theory, a theory of psychiatric nursing that advocates that such intervention promotes an important bond with the user and encourages coping and management tools in anxiety situations, being a basic component in the training of generalist nurses^(29)^. According to Cofen Resolution No. 678/2021, which approves the work of the Nursing Team in Mental Health and Psychiatric Nursing, it is the nurse’s responsibility to provide individual or group emotional care using psychotherapy techniques. However, it establishes that, to work in such a team, the nurse should preferably have a postgraduate degree in Mental Health, Psychiatric Nursing, or Psychosocial Care^(25, 30)^. Thus, considering the PHC nurses, who, for the most part, do not have a specialization in mental health, the results of the present study are to some extent expected. However, it is important to emphasize that the offer of groups in PHC is recommended by Booklet 34^(11)^, and is therefore possible for the nurse to carry out, and that, if implemented, it can favor primary care, that is, promote and prevent mental health problems in the population. In this case, it is important to emphasize the importance of ensuring that groups do not favor the sharing of care, trivializing its implementation only to meet the demands of organizations.

Regarding the application of scales, although most understand it as a nursing responsibility and consider it feasible to implement it in PHC units, only one third of nurses feel qualified to perform them. It is understood that scales are important in the process of diagnosis and implementation of care^(31)^. The use of these scales by nurses is supported by Opinion No. 036/2019 of Cofen, as long as they are not exclusive to other professionals. Their application allows for more appropriate proposals for the treatment of users, and it is essential for nurses to understand the impact of an assessment of mental state for planning their actions^(32)^. Therefore, this activity is shown as a possibility to facilitate mental health care for nurses, since the prior identification of such issues can facilitate the management of care, with better direction and decision-making.

The PHC, as the user’s gateway to the Psychosocial Care Network (PSCN), involves the transfer of cases to other professionals and/or other specialized services. The collaborative care model, which has been emphasized worldwide, describes the process by which PHC and mental health professionals share resources, experiences, knowledge and decision-making, through the inclusion of a care manager, communication and care centered on the person, family and community, in order to guarantee access to individuals seeking mental health care in the PHC^(4)^. This model aims to overcome the fragmentation of care, based on interdisciplinary techniques, and in Brazil, it is implemented in PHC services based on matrix support in health, which aims to ensure specialized support for teams and professionals responsible for caring for health problems, in a personalized and interactive manner^(33, 34)^. It operates with the concept of core and field. Thus, a specialist with a certain core of training supports specialists with another core, with the objective of increasing the effectiveness of their performance^(33)^.

The challenges for the consolidation of matrix support in mental health in PHC were identified in a previous study that highlighted the bureaucratization of the flow between services; the lack of clarity among professionals about their role; the high turnover of professionals in both PHC and specialized services; the lack of training; the stigma related to mental health care, among others, emphasizing that such factors perpetuate the logic of referral to the detriment of the real meaning of matrix support^(34)^. In addition to such challenges, it is understood that the scrapping and overload to which PHC is subjected hinder the improvement of the practice of matrix support and favor the perpetuation of the logic of referral, as professionals do not feel capable of absorbing this demand for care. However, such factors were not raised in the present study, which indicates the need for future research that covers these issues.

Regarding Advanced Practices in Rehabilitation, more than half of the nurses did not consider themselves fully qualified to perform such interventions. Psychosocial Rehabilitation is understood as actions that aim to increase the capacity for affective, social and economic contractuality, to enable the best possible level of autonomy for life in the community for people suffering from severe and persistent mental disorders. It is achieved through Yesple psychoeducational interventions and, mainly, through the intermediation of intersectoral actions, which is why the work of PHC professionals is so important^(11)^.

Because it is a concept originally aimed at the care of people with severe and persistent mental disorders, it can make professionals feel insecure about feeling truly qualified to perform such work. However, it is understood that Psychosocial Rehabilitation encompasses relational, contractual and everyday issues that can be worked on for any individual. In short, their approach by generalist nurses could be related to the way these contents are presented in the training of generalist nurses based on aspects of interpersonal relationships, empathy, welcoming, active listening and therapeutic communication, which would not necessarily depend on advanced training in mental health through specializations and/or postgraduate studies. In this sense, it is understood that there is a gap that needs to be further investigated regarding the mental health content to which these professionals are being exposed in their undergraduate courses, which opens up possibilities for future research.

Most participants identified social and family support as being within their attributions, possible to perform and felt qualified for such activity. Although they were listed in the specific component of mental health care, it is understood that they are not restricted to this context, especially considering that PHC works from the perspective of comprehensive care for the individual, encompassing social and family support is essential regardless of the diagnosis^(11)^. It is important to note that the methodological option defined in this study was based on the perspective of Psychosocial Rehabilitation, which, as previously mentioned, encompasses relational issues.

The Singular Therapeutic Project (STP) is seen by almost all nurses as possible to implement in PHC, however, almost half do not feel fully qualified to implement it. This result should be viewed with greater attention, as the STP aims at the individuality of the subject and its actions are not restricted to clinical problems or pharmacological interventions. In addition, it considers vulnerability and values cultural, economic and social spheres, in order to provide integrated and shared care with the team, the user and the community, which favors the maintenance and quality of the care provided^(35)^. Considering these factors, although the STP is not mandatory for all cases in PHC, its use by nurses is essential for more complex cases, since it systematizes important issues considering the comprehensive care of the subject.

Patient safety, through guidelines on the rational use of psychotropic drugs, is recognized as a responsibility by nurses, and its practice in PHC is validated, with just over half of them feeling qualified to perform it. Studies have found that nurses provide care based on the search for prescription renewals, which highlights the medicalizing factor that is rooted in the concept of mental health care^(4, 15)^. This perception still prevails, even with the transition from the hospital-centered paradigm to person-centered care and psychosocial rehabilitation. This highlights the need for future studies that investigate which attitudinal aspects of professionals should be better developed during their training, in addition to theoretical and procedural content.

The activities described in component III, health and continuing education, yield a worrying result, since a significant fraction of nurses do not feel qualified to perform such actions focused on mental health and psychiatry, even reporting their feasibility, corroborating findings from previous studies^(36, 37, 38)^.

Health education is related to educational activities involving staff, users/family members/community, and can occur in different social facilities, such as schools, social organizations and religious institutions^(36)^. Nurses, as educators, can provide strategies that will enhance nursing care, with a view to promoting the physical and mental health of users, which encourage behavioral changes to improve quality of life through discussions and reflections, using resources available in health services or innovative strategies^(36, 39)^.

Continuing education, on the other hand, involves the construction of knowledge based on the realities faced in daily life and the health needs of the population, envisioning the transformation of professional practices and the work process^(40)^. This action is guaranteed by the National Policy for Continuing Education (PNEPS), institutionalized through Ordinance No. 198/GM, of February 13, 2004, which seeks to encompass the responsibility of the Unified Health System to be the organizer of the training of human resources in the health area^(41)^. In this sense, it is understood that such tools are fundamental for the enhancement of mental health care by PHC nursing professionals.

It is observed that nurses recognize the importance of the activities and their responsibility regarding the care of people with needs related to mental health, due to the essence of the profession and the law of professional practice. However, the identity of this practice is influenced by the lack of clarity of their role and function in the work articulated with multidisciplinary teams and with their private activities. As a reference professional, this recognition is fundamental for professional autonomy, which corroborates studies that report difficulties of nurses in dealing with specific mental health issues, which generate insecurity in users^(5, 37)^.

Regarding the feasibility of actions, both in the context of universal and specific actions, most nurses emphasize that the actions can be carried out in the PHCU. However, studies show that the actions of nurses related to mental health in the PHC are specific, generally restricted to identifying demands and referring patients to the secondary network, which reduces care for patients undergoing drug therapy^(5, 37)^.

These aspects highlight the importance of reflecting on strategies to strengthen nursing professionals with regard to their training focused on mental health care. It is considered that the fundamental elements of primary mental health care — such as interpersonal relationships, therapeutic communication, welcoming, empathy, and qualified listening — constitute essential relational components. These elements should be part of the training of generalist nurses who will work in the PHC, which, ultimately, plays the role of organizing mental health care within the scope of the PSCN.

### Study limitations

The limitations of the study include the fact that issues related to the use of psychoactive sPHCUtances were not explored and that aspects of the perceptions of the professionals approached in a qualitative interview could contribute to a critical analysis of the data. Furthermore, it is understood that, since this is a descriptive study, the cause and effect of the aspects observed were not evaluated. It is also worth noting that the data must be interpreted in light of the references used to classify/group the practices, in this case, the systematic reviews, the guides and the national professional legislation selected by the researchers, which, in itself, is a limitation, given that such selection was influenced by the prior knowledge and subjectivity of each one, that is, other researchers could have had different perspectives at the time of selection.

### Contributions to the Nursing Field

This study contributes in several aspects. Firstly, based on the literature review, it is understood that it is one of the first published studies that has focused on listening to nurses about their perceptions and the identification of mental health practices in the PHC setting. Furthermore, the results found can support ongoing education actions for PHC nurses, based on questioning practices, collective construction and knowledge sharing, in order to enable interventions that they already identify as being their responsibility and possible to implement in the PHC setting. In addition, it contributes to triggering a discussion about undergraduate nursing curricula with the aim of emphasizing aspects of mental health care.

## CONCLUSIONS

It was found that nurses recognize mental health practices in their daily care, perform them and understand them as being within the scope of their actions; however, there is still a perception that they do not feel qualified to carry out such practices. Nurses feel more qualified to carry out universal actions associated with component I, when compared to component II, which is specific to mental health activities. Not only does the feasibility of carrying them out decrease, but reports of reduced training increase. In component III, the perception of a lack of training for continuing education deserves to be highlighted, since it involves actions to change care practices and work processes.

Therefore, training actions in mental health, post-training and during nursing training, become essential in the process of strengthening PHC as a gateway to the health demands of the population, as well as managerial adjustments in services, with better distribution of tasks, so that nursing professionals can dedicate themselves to individualized, humanized and comprehensive care for users with needs related to mental health, with safety in care and in the transition of care with the specialized network.
