# Supplementary material for: Nurses’ perception on mental health interventions in Primary Health Care
Source: Rev Bras Enferm. 2025 Sep 8;78(3):e20240260. doi: 10.1590/0034-7167-2024-0260 (PMC12419778; doi:10.1590/0034-7167-2024-0260)
Supplement: Translation

# Translation: Percepção de enfermeiros sobre intervenções em saúde mental na Atenção Primária à Saúde

**DOI:** 10.1590/0034-7167-2024-0260pt

**Authors:** Rafaela Sales Medeiros, Divane de Vargas, Jaqueline Lemos de Oliveira

**Affiliations:** I Universidade de São Paulo. São Paulo, São Paulo, Brasil

**Keywords:** Atenção Primária à Saúde, Enfermagem, Saúde Mental, Psiquiatria, Intervenção Psicossocial

## Abstract

**Objetivos:**

verificar a percepção de enfermeiros da Atenção Primária à Saúde acerca de intervenções em saúde mental empregadas em seu cotidiano de trabalho.

**Métodos:**

estudo quantitativo, descritivo-exploratório, desenvolvido em 66 Unidades Básicas de Saúde. A amostra constituiu-se de 239 enfermeiros. Para a análise descritiva, de acordo com as intervenções preconizadas nas diretrizes nacionais e com revisões sistemáticas, as práticas foram classificadas em três componentes: prática universal (I), específica em saúde mental (II) e práticas educacionais e universais (III).

**Resultados:**

em relação ao componente I, a maioria dos participantes consideraram todos os itens como de sua atribuição e possíveis de realizar, apenas para o item Práticas Integrativas e Complementares, a maioria dos participantes não se considerou capacitada. Dentre as atividades específicas de saúde mental, os itens relacionamento interpessoal terapêutico, práticas avançadas em reabilitação, grupos terapêuticos e aplicação de escalas foram aquelas para as quais os enfermeiros menos se consideraram capacitados para realizar. Sobre o componente III, educação permanente, foi o item para o qual apenas 40% dos enfermeiros se consideravam capacitados para realizar.

**Conclusões:**

verificou-se que os enfermeiros reconhecem as práticas de saúde mental no atendimento cotidiano do serviço, assim como as executam e entendem como sendo do escopo de suas ações, entretanto ainda há a percepção de não se sentirem capacitados para a realização de tais práticas.

## INTRODUÇÃO

Dados da Organização Mundial de Saúde (OMS) estimam que cerca de 720 milhões de pessoas possuam necessidades relacionadas à saúde mental, como transtornos mentais, neurológicos e por uso de álcool e de outras drogas, sendo tais condições responsáveis por incapacidades e por grande carga de doenças em todo o mundo^(1)^. No Brasil, em 2019, os transtornos mentais (TM) causaram 22% de anos de vida ajustados por incapacidade (DALYs) e 38% de todos os anos vividos com incapacidade (YLDs), sendo os distúrbios mais comuns para essas perdas os transtornos depressivos, os ansiosos, a bipolaridade e a esquizofrenia^(2)^. Além disso, o país ocupa o quarto lugar entre os países latino-americanos com o maior aumento anual de suicídios^(3)^.

De acordo com a OMS, 20% dos atendimentos na Atenção Primária à Saúde (APS) estão relacionados aos TMs, o que reforça a importância desses cenários de profissionais que atuam para o cuidado em saúde mental da população^(4)^. Inversamente aos indicadores epidemiológicos, evidencia-se a insuficiência de profissionais especializados para cuidar da saúde mental no Brasil e no mundo que, somada à escassez de ações e à falta de capacitação dos profissionais, incluindo os enfermeiros, desvaloriza o serviço para o enfrentamento desse panorama^(5, 6, 7)^.

Dentre as razões apontadas na literatura para as dificuldades dos enfermeiros da APS em se envolver com as demandas de saúde mental estão: a ausência de estratégias e de instrumentos para o atendimento na área, a falta de capacitação e as inseguranças em lidar com situações específicas na prática cotidiana^(5, 6, 7, 8)^.

Esses dados resultam em uma ação limitada do enfermeiro em realizar o acolhimento e em identificar os sintomas de adoecimento mental, o que interfere diretamente na prática assistencial, que, por fim, direciona o cuidado para outros profissionais da equipe ou para serviço especializado, sem a utilização de intervenções em saúde mental que são específicas do profissional enfermeiro e já preconizadas nos *guidelines* nacionais e em regulamentações profissionais^(5, 6, 7, 8)^.

## OBJETIVOS

Verificar a percepção de enfermeiros da APS acerca de intervenções em saúde mental empregadas em seu cotidiano de trabalho.

## MÉTODOS

### Aspectos éticos

Este estudo foi aprovado pelo Comitê de Ética e Pesquisa da Instituição sede. O consentimento informado foi obtido de todos os sujeitos mediante assinatura prévia do Termo de Consentimento Livre e Esclarecido.

### Desenho, período e local do estudo

Estudo descritivo exploratório com recorte transversal norteado pela ferramenta STROBE. Realizado entre março e agosto de 2023, em Unidades Básicas de Saúde (UBS) tradicionais (sem Estratégia Saúde da Família), no Município de São Paulo^(9)^.

### População e amostra

Com vistas a recrutar uma amostra representativa dos enfermeiros e a minimizar o viés de seleção, utilizou-se amostragem por estratificação em subgrupos homogêneos. O cálculo amostral considerou uma margem de erro de 5%; recusas e/ou perdas em aproximadamente 10%; fatores de controle de confusão em 20%, e nível de confiança de 95%, o que resultou em uma amostra mínima de 199 enfermeiros; a amostra final constituiu-se de 239 enfermeiros. Os critérios de inclusão foram: ser enfermeiro da unidade por um período mínimo de 6 meses (justificado como período de adaptação e de integralização do profissional à equipe e às vivências referentes ao processo de saúde-doen-ça-adoecimento dos usuários) e já ter atendido pessoas com necessidades relacionadas à saúde mental. O critério de exclusão foi ter alguma condição crônica de saúde que inviabilizasse a participação na pesquisa.

### Coleta de dados

Para a coleta de dados, utilizou-se o instrumento criado pelos autores, com questões de múltipla escolha com respostas simples e objetivas, para minimizar o viés de resposta, distribuídas em três categorias: I - identificação e dados sociodemográficos (15 questões); II - perfil da formação acadêmica e assistencial (14 questões). Neste item, foi questionada a frequência de atendimentos a pessoas com queixas relacionadas a transtornos persistentes, como depressão, ansiedade e esquizofrenia, bem como a transtornos mentais comuns, caracterizados por sintomas somáticos não psicóticos que geram incapacidade funcional, tais como tristeza, fadiga, desânimo, alterações no sono e no apetite, dores, tonturas ou mesmo distúrbios gástricos e intestinais^(10)^; III - Questionário referente à atuação do enfermeiro (20 questões). Essa última categoria inclui intervenções selecionadas do Caderno 34 da Atenção Básica, elaborado pela Secretaria de Atenção à Saúde, em parceria com o Ministério da Saúde, o qual compartilha conhecimentos específicos e apresenta ferramentas e estratégias de intervenções terapêuticas para o cuidado em saúde mental^(11)^; bem como da resolução COFEN 599/2018, que dispõe sobre as ações generalistas e específicas de saúde mental que competem ao enfermeiro^(12)^; além de uma revisão de escopo que descreve as intervenções de enfermagem em saúde mental na APS^(6)^, destacando-se a importância da apropriação dessas ferramentas para o cuidado.

As categorias foram classificadas em três componentes elaborados com base nas diretrizes da atenção básica do trabalho em equipe^(9)^ sendo eles: Componente I - Intervenções de prática universais, abrangendo intervenções que possuem interface com outras linhas de cuidado da APS; Componente II - Intervenções de prática específica de saúde mental e de psiquiatria; e Componente III - Intervenções baseadas em atividades educativas e gerenciais, relacionadas ao preparo da equipe de enfermagem para a atuação frente às situações de saúde mental e registro. Tais categorias respaldaram a interpretação dos dados.

No município de São Paulo, no período da coleta, havia 392 UBS registradas, distribuídas em seis Coordenadorias Regionais de Saúde (CRS). Para atender à amostra por região, foram selecionadas unidades conforme a disponibilidade, tanto dos gerentes como da equipe de coleta, sendo incluídas 17 unidades na CRS Sudeste; 02 unidades na CRS Centro; 16 unidades na CRS Leste; 12 unidades na CRS Norte; 08 unidades na CRS Oeste e 11 unidades na CRS Sul, totalizando 66 UBS. As entrevistas foram agendadas previamente e a coleta se deu presencialmente nos locais de trabalho dos participantes. Os dados foram armazenados e gerenciados por meio de formulários da ferramenta eletrônica de captura de dados REDCap. A duração média das entrevistas foi de 25 minutos.

### Análise dos dados

Para a análise dos dados, utilizou-se o R Development Core Team, versão 4.1.0, onde foram sistematizadas as tabelas de distribuição de frequência para a apresentação dos achados, sendo aplicada análise estatística simples e descritiva.

## RESULTADOS

### Caracterização sociodemográfica e profissional da amostra

A amostra foi composta por 239 enfermeiros majoritariamente do sexo feminino (88,2% n=211); com idade média de 40 anos (SD11,2); casadas (55,6% n=133); com tempo de atuação na unidade de 1 a 4 anos (45,4% n=108) e jornada de trabalho integral (75,3% n=180). O tempo médio de formação foi de 12 anos (DP = 5,25) e a maioria era de instituições privadas (82,4% n=197). Com relação ao nível educacional, 95,2% (n=210) cursaram especialização, e a especialidade mais frequente foi estratégia de saúde da família (39,4% n=86), seguida de saúde pública (38,9% n=85). Com relação ao preparo para atuar na saúde mental e na psiquiatria, a maioria dos entrevistados (91,6% n=164) informou somente o preparo na graduação, oferecido por meio de aulas expositivas.

### Frequência de atendimentos de saúde mental

Quando questionados sobre a frequência de atendimentos de saúde mental, a ansiedade (71%) e a depressão (61,5%) foram as mais relatadas pelos enfermeiros, seguidas pelo transtorno mental comum atendido diariamente por 54,8% dos enfermeiros (Figura 1).

**Figura 1:** Frequência dos atendimentos por quadro clínico na UBS

### Componente I - Intervenções de práticas universais

Dentre as práticas universais, aquelas direcionadas a todos os usuários que buscam por cuidado em saúde na APS, incluindo as pessoas com necessidades relacionadas à saúde mental, as respostas dos enfermeiros, relacionadas ao reconhecimento de atribuição, à possibilidade de realização e à percepção da capacitação estão evidenciadas na Tabela 1.

**Tabela 1:** Distribuição das respostas dos participantes ao componente I de práticas assistenciais universais quanto ao reconhecimento como uma prática de sua responsabilidade, possibilidade de realização e percepção sobre a própria capacitação para a utilização na prática cotidiana no cenário da Atenção Primária à Saúde, São Paulo, São Paulo, Brasil, 2023 (N=239)

Intervenção	Considera ser sua atribuição	n(%)	Possível de realizar no cenário da APS	n(%)	Considera-se capacitado	n(%)
Acolhimento	Sim	230(96,6)	Sim	224(93,7)	Totalmente	134(56)
	Não	6(2,5)	Não	9(3,7)	Parcialmente	93(38,9)
	Não estou certo	2(0,8)	Não estou certo	6(2,5)	Não se considera	12(5,0)
Triagem	Sim	209(95,0)	Sim	219(99,5)	Totalmente	189(85,9)
	Não	10(4,5)	Não	1(0,4)	Parcialmente	30(13,6)
	Não estou certo	1(0,4)	Não estou certo	19(7,9)	Não se considera	1(0,4)
Consulta de Enfermagem	Sim	223(93,3)	Sim	217(90,8)	Totalmente	153(64)
	Não	9(3,7)	Não	13(5,4)	Parcialmente	72(30,1)
	Não estou certo	7(2,9)	Não estou certo	9(3,7)	Não se considera	14(5,8)
Acompanhamento pré-natal	Sim	228(95,4)	Sim	224(93,7)	Totalmente	150(62,7)
	Não	7(2,9)	Não	6(2,5)	Parcialmente	83(34,7)
	Não estou certo	4(1,6)	Não estou certo	9(3,7)	Não se considera	6(2,5)
Atendimento domiciliar	Sim	211(88,2)	Sim	206(86,1)	Totalmente	131(54,8)
	Não	17(7,1)	Não	18(7,5)	Parcialmente	91(38,0)
	Não estou certo	11(4,6)	Não estou certo	15(6,2)	Não se considera	17(7,1)
Práticas integrativas e complementares	Sim	181(75,7)	Sim	205(85,7)	Totalmente	56(23,4)
	Não	45(18,8)	Não	25(10,4)	Parcialmente	54(22,5)
	Não estou certo	13(5,4)	Não estou certo	9(3,7)	Não se considera	129(53,9)
Gerência dos casos	Sim	216(90,3)	Sim	223(93,3)	Totalmente	142(59,4)
	Não	16(6,6)	Não	11(4,6)	Parcialmente	82(34,3)
	Não estou certo	7(2,9)	Não estou certo	5(2,0)	Não se considera	15(6,2)

### Componente II - Intervenções de prática específica de saúde mental e psiquiatria

Dentre as práticas específicas de saúde mental, relacionadas ao cuidado a pessoas com necessidades relacionadas à saúde mental, as respostas dos enfermeiros referentes ao reconhecimento de atribuição, à possibilidade de realização e à percepção da capacitação estão evidenciadas na Tabela 2.

**Tabela 2:** Distribuição das respostas dos participantes ao componente II de prática específica de Saúde Mental e Psiquiatria quanto ao reconhecimento como uma prática de sua responsabilidade, com possibilidade de realização e percepção sobre sua capacitação para utilização na prática cotidiana no cenário da Atenção Primária à Saúde, São Paulo, São Paulo, Brasil, 2023 (N=239)

Intervenção	Considera ser sua atribuição	n(%)	Possível de realizar no cenário da APS	n(%)	Considera-se capacitado	n(%)
Aconselhamento	Sim	212(88,7)	Sim	222(88,7)	Totalmente	121(50,6)
	Não	21(8,7)	Não	9(3,7)	Parcialmente	104(43,5)
	Não estou certo	6(2,5)	Não estou certo	6(2,5)	Não Se considera	14(5,8)
Relacionamento Interpessoal Terapêutico	Sim	191(79,9)	Sim	182(76,4)	Totalmente	80(33,4)
	Não	28(11,7)	Não	39(16,3)	Parcialmente	111(46,4)
	Não estou certo	20(8,37)	Não estou certo	17(7,14)	Não Se considera	48(20,0)
Apoio Social	Sim	227(94,9)	Sim	219(91,6)	Totalmente	143(60,0)
	Não	11(4,6)	Não	10(4,1)	Parcialmente	83(34,8)
	Não estou certo	1(0,4)	Não estou certo	10(4,1)	Não Se considera	12(3,3)
Apoio familiar	Sim	212(88,7)	Sim	211(88,2)	Totalmente	137(57,3)
	Não	15(6,8)	Não	20(8,3)	Parcialmente	85(35,5)
	Não estou certo	12(5,0)	Não estou certo	8(3,3)	Não Se considera	17(7,1)
Práticas Avançadas em Reabilitação	Sim	163(68,2)	Sim	172(71,9)	Totalmente	66(27,6)
	Não	61(25,5)	Não	49(20,5)	Parcialmente	87(36,4)
	Não estou certo	15(6,2)	Não estou certo	18(7,5)	Não Se considera	86(35,9)
Segurança do usuário relacionado ao uso de psicofármacos	Sim	187(78,2)	Sim	209(87,4)	Totalmente	128(53,5)
	Não	39(16,3)	Não	19(7,9)	Parcialmente	79(33,0)
	Não estou certo	13(5,4)	Não estou certo	11(4,6)	Não se considera	32(13,3)
Encaminhamentos e apoio matricial	Sim	219(91,6)	Sim	232(97,0)	Totalmente	154(64,7)
	Não	12(5,0)	Não	4(1,6)	Parcialmente	76(31,9)
	Não estou certo	8(3,3)	Não estou certo	3(1,2)	Não Se considera	8(3,3)
Projeto Terapêutico Singular	Sim	217(91,1)	Sim	212(97,0)	Totalmente	137(57,3)
	Não	13(5,4)	Não	17(7,1)	Parcialmente	80(33,4)
	Não estou certo	8(3,3)	Não estou certo	10(4,1)	Não Se considera	22(9,2)
Grupos Terapêuticos	Sim	178(74,4)	Sim	201(84,1)	Totalmente	66(27,6)
	Não	44(18,4)	Não	23(9,6)	Parcialmente	118(49,3)
	Não estou certo	17(7,1)	Não estou certo	15(6,2)	Não Se considera	55(23,0)
Aplicação de Escalas	Sim	169(70,7)	Sim	181(75,7)	Totalmente	80(33,4)
	Não	45(18,8)	Não	35(14,6)	Parcialmente	69(28,8)
	Não estou certo	25(10,4)	Não estou certo	23(9,6)	Não Se considera	90(37.6)

### Componente III - Intervenções baseadas em atividades educativas e gerenciais

Dentre as atividades educativas e gerenciais, as respostas dos enfermeiros relacionadas ao reconhecimento de atribuição, à possibilidade de realização e à percepção da capacitação estão evidenciadas na Tabela 3.

**Tabela 3:** Distribuição das respostas dos participantes ao componente III - Intervenções baseadas em atividades educativas e gerenciais quanto ao reconhecimento como uma prática de sua responsabilidade, possibilidade de realização e percepção sobre a própria capacitação para a utilização na prática cotidiana no cenário da Atenção Primária à Saúde, São Paulo, São Paulo, Brasil, 2023 (N=239)

Intervenção	Considera ser sua atribuição	n(%)	Possível de realizar no cenário da APS	n(%)	Considera-se capacitado	n(%)
Educação em Saúde	Sim	211(88,2)	Sim	222(92,8)	Totalmente	125(52,3)
	Não	21(8,7)	Não	12(5,0)	Parcialmente	89(37,2)
	Não estou certo	7(2,9)	Não estou certo	5(2,0)	Não se considera	25(10,4)
Educação Permanente	Sim	208(87,0)	Sim	207(86,6)	Totalmente	96 (40,1)
	Não	23(9,6)	Não	24(10,0)	Parcialmente	103(43,1)
	Não estou certo	8(3,3)	Não estou certo	8(3,3)	Não se considera	40(16,7)
Registro	Sim	233(97,4)	Sim	235(98,3)	Totalmente	199(83,2)
	Não	5(2,0)	Não	3(1,2)	Parcialmente	34(14,2)
	Não estou certo	1(0,4)	Não estou certo	1(0,4)	Não se considera	6(2,5)

## DISCUSSÃO

Os resultados do estudo sugerem que os enfermeiros reconhecem as intervenções em saúde mental e as identificam como atividades de sua responsabilidade, atendendo, em sua prática, pessoas com necessidades relacionadas à saúde mental e executando as intervenções descritas; a viabilidade também é relatada na execução como possível na APS. O resultado reflete a atuação do enfermeiro como relevante no atendimento à saúde mental, pois é o profissional da linha de frente da assistência cuja atuação vai além do cuidado físico, integrando esferas biopsicossociais, econômicas e ambientais, a fim de assegurar uma assistência eficaz e integral ao indivíduo^(7)^. Esses são aspectos essenciais, considerando-se a APS, que se baseia no modelo assistencial centrado na pessoa, na família, no estabelecimento de vínculos, na integralidade e longitudinalidade, o que supera o modelo biomédico^(13, 14, 15)^.

Em relação ao componente I, constituído pelas atividades universais, a maioria dos participantes considerou todos os itens como de sua atribuição e possíveis de realizar no cenário da APS, no entanto, quanto ao item Práticas Integrativas e Complementares (PICS), a maioria não se considerou capacitada para realizar tal intervenção. O Conselho Federal de Enfermagem (Cofen), através da Resolução 739/24, regulamentou a atuação da enfermagem nas PICS. Nesse sentido, profissionais devidamente capacitados têm autonomia para atuar com tais intervenções^(16)^. Por se tratar de uma resolução recente, entende-se que é necessário o incentivo por parte dos serviços para que os profissionais da enfermagem se capacitem e tenham essas intervenções como estratégias de cuidado na APS, uma vez que estimulam a perspectiva integral e multiprofissional do cuidado, ao possibilitar maior adesão dos pacientes aos tratamentos^(17)^. Contudo, cabe salientar que a disponibilidade tanto de recursos humanos, quanto de materiais são itens que limitam a ampliação do acesso a esse cuidado, tendo em vista resultado de estudo prévio^(18)^. Nesse sentido, esses são fatores que devem ser considerados na implementação de políticas que incentivem tal cuidado.

Destaca-se que o acolhimento foi o item que mais profissionais identificaram como sendo de sua atribuição. Estudo de revisão aponta que tal intervenção é a principal tecnologia do cuidado e está dentro do rol de habilidades do profissional da enfermagem^(6)^. Entende-se que é uma intervenção relacional, essencial para a criação de vínculo, que promove o cuidado integral e direcionado^(15)^. Considerando o atendimento em saúde mental, essa ação facilita a identificação de sinais, de sintomas e de possíveis desencadeantes do transtorno e, consequentemente, o direcionamento do fluxo adequado de atendimento^(19)^. O acolhimento está descrito nas diretrizes e nas funções dos enfermeiros em *guidelines* nacionais^(9, 12)^, sendo, portanto, uma importante ferramenta para o cuidado em saúde mental na APS^(20)^.

Segundo as diretrizes da APS, o acolhimento e a triagem são fatores importantes no processo de responsabilização da equipe pelo usuário. Nesse sentido, o uso da escuta qualificada e a busca da atenção integral garantem uma atenção resolutiva e responsável. Os desdobramentos observados durante as consultas de enfermagem favoráveis aos cuidados com os usuários são potencializados com o atendimento individualizado, ao promover ações articuladas com a equipe multiprofissional de prevenção, de sinalização de riscos e de promoção da saúde^(8, 21, 22)^. Autores apontam a capacitação como ferramenta para a qualidade dos atendimentos, bem como a necessidade de protocolos claros que orientem a prática^(23, 24, 25)^.

O processo de trabalho do enfermeiro é marcado pelo acúmulo de tarefas assistenciais (atendimento ao usuário, à família e à comunidade nos contextos individuais e coletivos) com as tarefas administrativas (gerenciamento de pessoas e processos administrativos). Esse aglomerado de tarefas é marcado pelo conflito entre praticar a assistência direta e realizar os processos administrativos, sendo que as cobranças relacionadas ao cumprimento das atividades administrativas não se equivalem às referentes à qualidade do atendimento de saúde ao usuário e à família^(23)^. Se, por um lado, esse acúmulo de tarefas pode prejudicar o cuidado ao usuário, por outro, entende-se que, como um profissional importante na atuação multiprofissional na APS, essa articulação é fundamental na transição de cuidados para os serviços especializados, pois, no contexto da gerência dos casos, articula as dimensões gerenciais e assistenciais, para identificar questões de saúde, promover ações assertivas e nortear o cuidado^(13, 14, 26, 27, 28)^.

Em relação às atividades do componente II, todas foram consideradas pela maioria dos participantes como sendo de sua atribuição e possíveis de realizar na APS. Entretanto, as atividades Relacionamento Interpessoal Terapêutico (RIT), a realização de grupos terapêuticos e a aplicação de escalas foram relatadas por mais da metade dos enfermeiros com pouca ou nenhuma capacitação para o exercício. Cabe destacar que o RIT se baseia nos conceitos da Teoria de Hildegard Peplau, teoria da enfermagem psiquiátrica que preconiza que tal intervenção promove um vínculo importante com o usuário e estimula ferramentas de enfrentamento e de manejo em situações de ansiedade, sendo componente básico na formação dos(as) enfermeiros(as) generalistas^(29)^.

De acordo com a Resolução Cofen Nº 678/2021 que aprova a atuação da Equipe de Enfermagem em Saúde Mental e em Enfermagem Psiquiátrica, cabe ao enfermeiro fazer atendimento emocional individual ou em grupo com a utilização de técnicas de psicoterapia, entretanto estabelece que, para atuar em tal equipe, o enfermeiro deverá, preferencialmente, ter pós-graduação em Saúde Mental, Enfermagem Psiquiátrica ou Atenção Psicossocial^(25, 30)^. Desse modo, pensando os enfermeiros da APS que, em sua maioria, não possuem especialização em saúde mental, os resultados do presente estudo são em certa medida esperados. Contudo, importa salientar que a oferta de grupos na APS é preconizada pelo Caderno 34^(11)^, sendo, então, possível de ser realizada pelo enfermeiro, e que, se implementada, pode favorecer o cuidado primário, ou seja, promover e prevenir agravos de saúde mental da população. Cabe salientar, neste caso, a importância de atentar-se para que os grupos também não favoreçam o compartilhamento do cuidado, banalizando sua realização apenas para atender as demandas das organizações.

No que tange à aplicação de escalas, apesar de a maior parte compreender como uma atribuição do enfermeiro e considerar viável a realização na APS, somente um terço dos enfermeiros se sentem capacitados para o exercício. Entende-se que as escalas são importantes no processo de diagnósticos e de implementação de cuidados^(31)^. O uso dessas escalas pelo enfermeiro é fundamentado pelo Parecer nº 036/2019 do Cofen, desde que não sejam privativas de outros profissionais. Sua aplicação permite propostas mais adequadas ao tratamento dos usuários, sendo fundamental o enfermeiro compreender o impacto de uma avaliação do estado mental para o planejamento de suas ações^(32)^. Sendo assim, tal atividade se mostra como uma possibilidade de facilitar, aos enfermeiros, o cuidado em saúde mental, uma vez que a identificação prévia de tais questões pode facilitar o manejo do cuidado, com melhor direcionamento e tomada de decisão.

A APS, como porta de entrada do usuário para a Rede de Atenção Psicossocial (RAPS), envolve a passagem de caso para outros profissionais e/ou para outros serviços especializados. O modelo de cuidado colaborativo, que tem sido enfatizado em todo o mundo, descreve o processo pelo qual os profissionais da APS e da saúde mental compartilham recursos, experiências, conhecimentos e tomadas de decisões, através da inclusão de um gerenciador do cuidado, da comunicação e do cuidado centrado na pessoa, na família e na comunidade, a fim de garantir o acesso aos indivíduos que buscam atendimento em saúde mental na APS^(4)^. Tal modelo objetiva superar a fragmentação do cuidado, baseando-se em técnicas interdisciplinares, sendo que, no Brasil, é implementado nos serviços de APS a partir do apoio matricial em saúde, que tem por finalidade assegurar retaguarda especializada a equipes e a profissionais encarregados da atenção a problemas de saúde, de maneira personalizada e interativa^(^33,34^)^. Opera com o conceito de núcleo e de campo. Assim, um especialista com determinado núcleo de formação apoia especialistas com outro núcleo, com o objetivo de ampliar a eficácia de sua atuação^(33)^.

Os desafios para a consolidação do apoio matricial em saúde mental na APS foram identificados em estudo prévio que destacou a burocratização do fluxo entre os serviços; a falta de clareza entre os profissionais sobre o seu papel de atuação; a alta rotatividade de profissionais tanto da APS quanto dos serviços especializados; a falta de capacitação; o estigma relacionado ao cuidado em saúde mental, entre outros, ressaltando que tais fatores perpetuam a lógica do encaminhamento em detrimento do real sentido do matriciamento^(34)^. Para além de tais desafios, entende-se que o sucateamento e a sobrecarga a que a APS está submetida dificultam o aprimoramento da prática do apoio matricial e favorecem a perpetuação da lógica do encaminhamento, pois os profissionais não se veem capazes de absorver mais essa demanda de cuidado. Entretanto, tais fatores não foram levantados no presente estudo, o que indica a necessidade de pesquisas futuras que abarquem essas questões.

Em relação às Práticas Avançadas em Reabilitação, mais da metade dos enfermeiros não se considerou totalmente capacitada para realizar tais intervenções. A Reabilitação Psicossocial é entendida como ações que visam aumentar a capacidade de contratualidade afetiva, social e econômica, para viabilizar o melhor nível possível de autonomia para a vida na comunidade das pessoas que sofrem com transtornos mentais graves e persistentes. É alcançada por meio de intervenções psicoeducacionais simples e, principalmente, pela intermediação de ações intersetoriais, sendo, por isso, tão importante a atuação dos profissionais da APS^(11)^.

Por ser um conceito originalmente voltado para o cuidado de pessoas com transtornos mentais graves e persistentes, pode gerar nos profissionais insegurança em se sentirem realmente capacitados para tal atuação. Contudo, entende-se que a Reabilitação Psicossocial abarca questões relacionais, contratuais e do cotidiano que podem ser trabalhadas para qualquer indivíduo. Sua abordagem pelos enfermeiros generalistas, em suma, poderia estar relacionada ao modo como esses conteúdos são apresentados na formação do enfermeiro generalista a partir de aspectos do relacionamento interpessoal, de empatia, de acolhimento, de escuta ativa e de comunicação terapêutica, o que não dependeria necessariamente de uma formação avançada em saúde mental por meio de especializações e/ou de pós-graduação. Nesse sentido, entende-se que há uma lacuna a ser mais investigada sobre o conteúdo de saúde mental a que estão sendo expostos tais profissionais na graduação, o que abre possibilidades para futuras pesquisas.

A maioria dos participantes identificaram o apoio social e familiar como sua atribuição, sendo possíveis de se realizar e se sentiam capacitados para tal atividade. Apesar de terem sido elencadas no componente específico dos cuidados em saúde mental, entende-se que não estão restritas a tal contexto, principalmente considerando que a APS trabalha na perspectiva do cuidado integral do indivíduo, abarcar o apoio social e familiar é imprescindível independente do diagnóstico^(11)^. Ressalta-se que a opção metodológica definida no presente estudo foi fundamentada na perspectiva da Reabilitação Psicossocial, a qual, como pontuado anteriormente, abrange as questões relacionais.

O Projeto Terapêutico Singular (PTS) é visto por quase a totalidade dos enfermeiros como possível de se realizar na APS, contudo quase metade não se sente totalmente capacitada para sua realização. Esse resultado deve ser visto com maior atenção, pois o PTS visa à individualidade do sujeito e suas ações não se restringem a problemas clínicos ou a intervenções farmacológicas. Além disso, considera a vulnerabilidade e valoriza as esferas culturais, econômicas e sociais, a fim de realizar um cuidado integrado e compartilhado com a equipe, com o usuário e com a comunidade, o que favorece a manutenção e a qualidade da assistência prestada^(35)^. Considerando tais fatores, apesar de o PTS não ser obrigatório para todos os casos na APS, entende-se como essencial seu uso pelo enfermeiro para casos mais complexos, uma vez que sistematiza questões importantes pensando o cuidado integral do sujeito.

A segurança do paciente, através de orientações do uso racional de psicotrópicos, tem sua responsabilidade reconhecida pelos enfermeiros, assim como validado seu exercício na APS, sendo que pouco mais da metade destes se sente capacitada para a realização. Estudos verificaram, entre os enfermeiros, atendimentos baseados na busca por renovação de receitas, o que evidencia o fator medicalizante que está arraigado na concepção de cuidado em saúde mental^(4, 15)^. Essa percepção ainda prevalece, mesmo com a transição do paradigma hospitalocêntrico para o cuidado centrado na pessoa e na reabilitação psicossocial. Isso evidencia a necessidade de estudos futuros que investiguem quais aspectos atitudinais dos profissionais devem ser mais bem desenvolvidos durante sua formação, além dos conteúdos teóricos e procedimentais.

As atividades descritas no componente III, de educação em saúde e permanente, trazem um resultado preocupante, já que uma fração significativa dos enfermeiros não se sente capacitada para realizar tais ações voltadas à saúde mental e à psiquiatria, mesmo relatando sua viabilidade, o que corrobora com achados de estudos prévios^(36, 37, 38)^.

A educação em saúde está relacionada às atividades educativas que envolvem equipe, usuários/familiares/comunidade, podendo ocorrer em diferentes equipamentos sociais, como escolas, organizações sociais e instituições religiosas^(36)^. O enfermeiro, como educador, pode abrir mão de estratégias que irão potencializar o cuidado de enfermagem, com vistas à promoção da saúde física e mental dos usuários, que estimulem mudanças de comportamento para melhorar a qualidade de vida a partir de discussões e de reflexões, com a utilização dos recursos disponíveis nos serviços de saúde ou em estratégias inovadoras^(36, 39)^.

Já a educação permanente envolve a construção de saberes a partir das realidades enfrentadas no cotidiano e das necessidades de saúde da população, vislumbrando a transformação das práticas profissionais e do processo de trabalho^(40)^. Tal ação é garantida pela Política Nacional de Educação Permanente (PNEPS), institucionalizada por meio da Portaria nº 198/GM, de 13 de fevereiro de 2004, a qual busca abarcar a responsabilidade do Sistema Único de Saúde de ser o ordenador da formação de recursos humanos na área da saúde^(41)^. Nesse sentido, entende-se que tais ferramentas são fundamentais para a potencialização do cuidado em saúde mental pelos profissionais de enfermagem da APS.

Observa-se que os enfermeiros reconhecem a importância das atividades e sua responsabilidade quanto ao atendimento às pessoas com necessidades relacionadas à saúde mental, pela essência da profissão e pela lei do exercício profissional, entretanto a identidade desse exercício sofre influências pela falta de clareza do seu papel e da função no trabalho articulado com equipes multidisciplinares e com suas atividades privativas. Como um profissional de referência, é fundamental esse reconhecimento para a autonomia profissional, o que corrobora estudos que relatam dificuldades dos enfermeiros em lidar com questões específicas de saúde mental, as quais geram insegurança aos usuários^(5, 37)^.

No que tange à viabilidade das ações, tanto no contexto das ações universais quanto das ações específicas, os enfermeiros enfatizam, em sua maioria, que as ações são possíveis de serem realizadas na UBS, entretanto estudos mostram que as ações de enfermeiros relacionadas à saúde mental na APS são pontuais, geralmente restritas à identificação de demandas e a encaminhamentos para a rede secundária, o que reduz o cuidado ao paciente em terapia medicamentosa^(5, 37)^.

Tais aspectos evidenciam a importância de refletir sobre estratégias para o fortalecimento dos profissionais de enfermagem no que diz respeito à sua formação voltada ao cuidado em saúde mental. Considera-se que os elementos fundamentais dos cuidados primários em saúde mental — como o relacionamento interpessoal, a comunicação terapêutica, o acolhimento, a empatia e a escuta qualificada — constituem componentes relacionais essenciais. Esses elementos devem integrar a formação do enfermeiro generalista que atuará na APS, a qual, em última análise, desempenha o papel de ordenadora do cuidado em saúde mental no âmbito da RAPS.

### Limitações do estudo

Entende-se como limitações do estudo: o fato de que não foram exploradas questões relacionadas ao uso de substâncias psicoativas e quais aspectos das percepções dos profissionais abordados em uma entrevista qualitativa poderiam contribuir para uma análise crítica dos dados. Além disso, entende-se que, por se tratar de um estudo descritivo, não se avaliaram causa e efeito dos aspectos observados.

Cabe salientar, ainda, que os dados devem ser interpretados à luz dos referenciais utilizados para a classificação/agrupamento das práticas, nesse caso, as revisões sistemáticas, os guias e as legislações profissionais nacionais selecionadas pelos pesquisadores – o que é uma limitação, tendo em vista que tal seleção foi influenciada pelo conhecimento prévio e pela subjetividade de cada um, ou seja, outros pesquisadores poderiam ter outros olhares no momento da seleção.

### Contribuições para a Área da Enfermagem

Este estudo contribui em diversos aspectos. Primeiramente, a partir do levantamento da literatura, entende-se que é um dos primeiros estudos publicados que tenha se ocupado de ouvir os enfermeiros sobre suas percepções e sobre a identificação das práticas de saúde mental no cenário da APS. Outrossim, os resultados encontrados podem basear ações de educação permanente aos enfermeiros da APS, a partir do questionamento da prática, da construção coletiva e do compartilhamento de saberes, a fim de viabilizar as intervenções que já foram identificadas como sendo de sua responsabilidade e possíveis de implementação no cenário da APS. Além disso, este trabalho contribui para desencadear uma discussão acerca dos currículos das graduações em enfermagem no intuito de enfatizar os aspectos do cuidado em saúde mental.

## CONCLUSÕES

Verificou-se que os enfermeiros reconhecem as práticas de saúde mental no atendimento cotidiano do serviço, executam e entendem como sendo do escopo de suas ações, entretanto ainda há percepção de não se sentirem capacitados para a realização de tais práticas. Os enfermeiros sentem-se mais capacitados para a realização das ações universais associadas ao componente I, quando comparadas ao componente II, sobre atividades específicas de saúde mental, não somente a viabilidade de realização diminui como também aumentam os relatos de diminuição da capacitação. No componente III, a percepção de falta de capacitação para educação permanente merece destaque, uma vez que envolve ações de mudanças de práticas de cuidado e processo de trabalho.

Portanto, ações de capacitação em saúde mental, pós-formação e durante a formação em enfermagem tornam-se imprescindíveis no processo de fortalecimento da APS como porta de entrada às demandas de saúde da população, assim como ajustes gerenciais nos serviços, com melhor distribuição de tarefas, para que o profissional enfermeiro consiga dedicar-se a uma assistência individualizada, humanizada e integral ao usuário com necessidades relacionadas à saúde mental com segurança no atendimento e na transição de cuidados com a rede especializada.
